# Dementia-Friendly Communities: The Role of Built Environment and Implementation Challenges in the U.S.

**DOI:** 10.1093/geroni/igaf122.143

**Published:** 2025-12-31

**Authors:** Xi Chen, Chanam Lee, Wenjin Wang

**Affiliations:** Texas A&M University, College Station, Texas, United States; Texas A&M University, College Station, Texas, United States; Texas A&M University, College Station, Texas, United States

## Abstract

The growing aging population has intensified public health concerns, particularly regarding dementia. Dementia-Friendly Communities (DFC) have gained global attention as a response to the increasing number of people living with dementia (PLWD) and the associated societal care burden. However, empirical research to inform policy briefs, design guidelines, and implementation strategies remains limited. This study explores expert perspectives on key neighborhood built environment features that support aging in place for PLWD and identifies challenges in implementing DFC in the U.S. A nationwide online expert survey was conducted from January to March 2023 and a total of 51 experts specializing in environment and aging research or practice provided complete responses and were included in data analysis. Quantitative ratings indicate that “land use & accessibility” and “safety” are the two most critical neighborhood-scale built environment domains for DFC design. Open-ended responses on environmental features highlight five key themes: (1) safety first, (2) clear space for better memory support, (3) balance between easy access and stimulation, (4) comfort by design with environmental context in mind, and (5) tailored design for different stages of dementia. Experts also identified financial challenges and knowledge barriers toward dementia or DFC as two major barriers to implementation in the U.S. By identifying these design priorities and implementation challenges, this study provides valuable insights to help policymakers, researchers, and design professionals better understand critical considerations for DFC design and facilitate the development of more feasible and effective solutions to promote dementia-friendly environments and support healthy aging in place.

